# Caries

**Published:** 1891-10

**Authors:** A. J. Holmes


ARTICLE IV.

### CARIES.

BY A. J. HOLMES, D. D. S., L. D. S.

When the source from which osseous structures derive their nourishment and vitality is destroyed, death follows as a necessary consequence.

This occurrence is called necrosis, or mortification of the bone, and sometimes authors speak of it as caries.

But, as caries of the bone is generally classified as a separate disease, I will speak on that subject later on in my paper. Necrosis may occur in any part of the body, but I will confine myself to that part most necessary to the dental surgeon: the oral cavity, and principally the alveolar.

The trouble may occur in the alveolar of either jaw, but is more liable to take place in the lower than in the upper. This, no doubt, is caused by the smaller blood and nerve supply in the lower jaw, thus weakening its vitality under that of the upper.

Though it is a plain fact that the alveolar process in either jaw, although like other bones, supplied with blood-vessels and nerves, their recuperative powers are weaker; and then to be deprived of a portion of its delicate, life-giving substance, by necrosis and exfoliation, or other causes, the injury is not so readily, as is often in other osseous tissues, repaired by the restoring efforts of nature. Again, necrosis may be confined to the socket of a single tooth, but more frequently it extends to several, and often through a portion of the alveolar border, and occasionally the entire alveolar, penetrating a part or the whole of the jaw.

When teeth are subjected to necrosed loosening, as is plainly shown by the natural necrosis that nature supplies for the expulsion of the temporary, they become dark by the destruction of their pulp, and are ultimately removed and fall out. Exceptionally, a number can be made to remain firm, and become useful.

Of all these cases the condition of the pulp-chamber of such teeth as have lost their pulps should receive prompt attention, to prevent the discharge of poisonous matter by way of the apical foramen, as such sources of irritation will, until it is removed, prevent a proper and perfect healing.

At first, the indications of trouble in the jaw that precede necrosis is not to be distinguished from maxillary or alveolar periostitis. The necrosed portion may be limited to bone in direct relation with inflamed periosteum, frequently excited by scurvy, syphilis, certain eruptive fevers, mercurialization and action of phosphorus, or, extending deeper, it may involve the entire alveolar border, and perhaps the palate and into the Highmore antrum, if it be the upper jaw affected. As it progresses, instead of confining itself to local or circumscribed swelling, the gums become congested and swollen over considerable area, unhealthy purulent pus oozes from the margin of the gums, detaching them from the bone, the teeth loosen, and in a few weeks the maxillary and alveolar plates also become disintegrated by the necrosed process. These pieces of bone lie dead and sequestrated, bathed in pus.

In all cases of necrosis the ingenuity of the operator must be depended upon to overcome the difficulties that may present themselves. Consequently, no set rules can be given to accomplish their treatment.

I, in the first place, generally remove the loose roots or teeth that are past redemption, then make free incisions into the swollen area to give relief to the blood-pressure, and give free vent to the pus that may be collected under the periosteum. Syringe the parts out with a warm solution of Listerine, peroxide of hydrogen and carbolic acid.

Too much stress cannot be placed on the remarkable value of Listerine in these affections.

When the patient is suffering great pain, instruct the use of heavy doses of antipyrine and Dover's powders, also call attention to the importance of keeping the bowels active.

Dismiss for further development. My method of treatment thus far will aid to stop periostitis and ostitis, and prevent necrosis, which is generally the result of continuous inflammation. But, in cases that are so far advanced, giving signs of disintegrated necrosed bone substance, I find the absorption and extrusion of the dead bone may usually be effected by the use of a ten per cent. solution of aromatic sulphuric acid, brought directly in contact with the area of the diseased part.

My method of using it is to inject through the fistulous openings, and directly in contact with the diseased bone, by means of a hypodermic with a blunt point, keeping it there for several minutes, followed by a washing of $H_2O_2$.

But, in extreme cases, where large sequestra are exfoliated and honey-combed carious bone is formed, I give assistance for their immediate relief by the use of the knife, aided by a four or a six per cent. solution of cocaine. Follow this by the weak sulphuric acid injections and mild washes, and in a short time we may generally dismiss our patient, cured.

But, before concluding the treatment of necrosis, and going to that of caries of the jaw, I wish to state that, after consulting many of our best authors as to the best treatment, I find that of Dr. J. T. Martin, to my opinion, a very good one. It is as follows:—

If seen in the early, active stage, give a good cathartic, say six to ten grains of calomel, followed in from four to eight hours by a large dose of saline cathartic, well diluted; follow this by ten to fifteen grains Dover's powder, or equivalent of some other opiate, to ally all pain, and repeat the dose as often as needed. If the skin is hot and dry, give drop doses of tincture of aconite every half hour.

in water, until the skin is moist and the action of the heart is modified.

By the action of these remedies the blood is diverted from the part, the pain checked, the irritation quieted, the force of the heart modified, and the general temperature lowered; in other words, the part put at rest.

He says it is of great importance to keep the bowels active wherever there is inflammation about the head or face. Lance the gum, with free incisions, down to the bone.

He has two reasons for this: 1st. Local blood-letting relieves the blood-pressure; and, 2nd, it gives vent to any pus collected, thus preventing extensive separation of the periosteum, and extensive necrosis in many cases.

He then advocates the painting of the gums and parts freely with tincture of iodine, hot fomentations applied over swollen parts, and similar remedies.

We can plainly see that his treatment so far is to combat periostitis and ostitis, and to prevent necrosis, caries or abscess, which are the results of continued inflammation.

He says that whenever necrosis of the lower jaw is discovered, the best method is to meet indications generally and locally, until the necrosed bone is separated from the living, and then assist nature in getting rid of the irritant by enlarging the opening and removing the sequestrum, or sequestra, as the case may be.

Keep the patient supported with remedies, as iron and bitter tonics. He also says that in these cases the supporting powers of quinine cannot be overestimated. Locally, the sinuses should be cleaned by stimulating alteratives, as solutions of iodine, Listerine, $H_2 O_2$, carbolic acid, etc., etc., which will bring about a cure, if careful attention be adhered to.

CARIES OF THE ALVEOLAR PROCESS AND MAXILLA.

When acute form is present, it is associated with inflammation of the gums and periosteum; periostitis being early observed.

It, like necrosis, is not very common, differing from the latter in being free from the odor, when kept cleaned, which characterizes necrosis. The causes are various, and one very common one is the pressure of the dead teeth and roots. Though ulceration and destruction of the tissues, resulting from syphilis or lupis, is one of its greatest causes. I have seen, on several occasions, caries in its most frightful forms, resulting from this affection, among the Indians of this country, where it has been led into the palate, destroying it until a terrible deformity followed, making a common cavity of the mouth and nose, and even involving the face to a great extent.

Caries, when well established in the maxilla, has one or more openings in the gum or neighboring parts. These canals or openings, in the majority of cases, are surrounded by fungus granulation. Harris states that in the early stage there is increased vascularity and congestion, which terminates in ulceration; the bone cells become enlarged by the breaking down of their walls, and filled with semi-organized lymph, the accumulation of which is attended with the rapid advance of the destructive process. While, according to Virchow the bone breaks up in its territories, the individual corpuscles undergo a new change (granulation and suppuration), and remnants composed of the oldest basis substance remain in the form of small, thin shreds, in the midst of the soft substance.

However, the whole process is a degenerative ostitis, in which the osseous tissue changes its structure, loses its chemical and morphological character, and so becomes a soft tissue, which no longer contains lime.

In treating caries, where it is not very extensive, I would first make an incision, expose the diseased part, and, if need be, I would at once pack them with iodoform gauze, or, what I think better, the boracic acid gauze, if it can be obtained, thus getting its antiseptic effects, and obviating the disagreeable odor that comes from the iodoform. Pack it, and dismiss the patient for a day or so, and, on his return, wash it with a warm solution of Listerine, and make an examination of the parts, so as to see how far the disease has gone, whether it has involved several roots or one root of the tooth or teeth, as it may be; then we know how far to proceed intelligently with our operations. The sense of touch will enable us to determine whether the bone is softened; we may, without the aid of vision, be able to make proper surgical operations, and not go beyond the territory involved by the diseases. I prefer a long, sharp bur, passing it into the osseous cavity, excising the ends of the roots of the tooth, and the caries bone, if thought necessary. Never fail to open each nerve cavity, and clean out the remaining debris at the proper time.

Why should we cut these roots off? Simply because they stand up there, and do not serve any useful purpose, and are often a source of irritation; they interfere with the formation of new tissue, consequently it will be far better to dispose of them. Having done this, we cleanse the cavity, as before directed. When the boracic gauze is not obtainable, the iodoform I believe to be better, if used with crystals of boracic acid, as they are dissolved more slowly than pulverized acid; they are more constant in their action—in other words, we retain the antiseptic agent longer by using this crystal which dissolves more slowly than the other agent.

Having packed accordingly, a few days later we make another ocular examination, and if we see little red granulations shooting up here and there over the surface of the part, it will soon be well, for we know it is an effort on the part of nature to close the cavity and effect a cure. In these cases, what I deem far better than fabric for the closure of the wounds, is wax. It may be softened and moulded to the cavities; remove the excess up the surface, and then replace. I do this because a wax plug is better than a fabric plug, and is more cleanly. It doesn't take up any secretions, and excite further irritation. I always, from time to time, remove the inner surface of the plug, to relieve it so that granulation may go on.

By-and-by the cavity will be filled to an extent which will prevent the retention of a plug longer, and the desired effect accomplished.—*Dominion Dental Journal.*

---

ARTICLE V.

### THE CARE OF THE DECIDUOUS TEETH.

